# A mass spectrometry imaging and lipidomic investigation reveals aberrant lipid metabolism in the orthotopic mouse glioma

**DOI:** 10.1016/j.jlr.2022.100304

**Published:** 2022-10-20

**Authors:** Hay-Yan J. Wang, Chiung-Yin Huang, Kuo-Chen Wei, Kuo-Chen Hung

**Affiliations:** 1 Department of Biological Sciences, National Sun Yat-Sen University, Kaohsiung, Taiwan; 2 Neuroscience Research Center, Chang Gung Memorial Hospital, Taoyuan, Taiwan; 3 Department of Neurosurgery, New Taipei Municipal TuCheng Hospital, New Taipei City, Taiwan; 4 Department of Neurosurgery, Chang Gung Memorial Hospital, Taoyuan, Taiwan; 5 School of Medicine, Chang Gung University, Taoyuan, Taiwan; 6 Department of Surgery, Kaohsiung Chang Gung Memorial Hospital, Kaohsiung, Chang Gung University College of Medicine, Taiwan

**Keywords:** tumor pathology, phospholipids, sphingolipids, MALDI MS imaging, unsupervised clustering analysis, LC-MS/MS, lipidomics, phosphatidylcholine, lipid-associated signaling pathways, ceramides, AKT, AKT serine/threonine kinase, CDase, ceramidase, Cer, ceramide, FADB, fatty acyl double bond, FADS2, FA desaturase 2, FC, fold change, IS, internal standard, LPC, lysophosphatidylcholine, LPE, lysophosphatidylethanolamine, LYPLA, lysophospholipase, MSI, MS imaging, PC, phosphatidylcholine, PE, phosphatidylethanolamine, PI, phosphatidylinositol, PI-PLC, phosphatidylinositol-specific PLC, PL, phospholipid, PLA_2_, phospholipase 2, SL, sphingolipid

## Abstract

Lipids perform multiple biological functions and reflect the physiology and pathology of cells, tissues, and organs. Here, we sought to understand lipid content in relation to tumor pathology by characterizing phospholipids and sphingolipids in the orthotopic mouse glioma using MALDI MS imaging (MSI) and LC-MS/MS. Unsupervised clustering analysis of the MALDI-MSI data segmented the coronal tumoral brain section into 10 histopathologically salient regions. Heterogeneous decrease of the common saturated phosphatidylcholines (PCs) in the tumor was accompanied by the increase of analogous PCs with one or two additional fatty acyl double bonds and increased lyso-PCs. Polyunsaturated fatty acyl-PCs and ether PCs highlighted the striatal tumor margins, whereas the distributions of other PCs differentiated the cortical and striatal tumor parenchyma. We detected a reduction of SM d18:1/18:0 and the heterogeneous mild increase of SM d18:1/16:0 in the tumor, whereas ceramides accumulated only in a small patch deep in the tumoral parenchyma. LC-MS/MS analyses of phospholipids and sphingolipids complemented the MALDI-MSI observation, providing a snapshot of these lipids in the tumor. Finally, the proposed mechanisms responsible for the tumoral lipid changes were contrasted with our interrogation of gene expression in human glioma. Together, these lipidomic results unveil the aberrant and heterogeneous lipid metabolism in mouse glioma where multiple lipid-associated signaling pathways underline the tumor features, promote the survival, growth, proliferation, and invasion of different tumor cell populations, and implicate the management strategy of a multiple-target approach for glioma and related brain malignancies.

Lipids, like proteins and carbohydrates, belong to a category of biological molecules that perform multiple important functions in the biological systems. Structurally, lipids form the cellular barriers to segregate the internal and external cellular environment and compartmentalize the intracellular space for specific functions (1). Metabolically, lipids are utilized as an important fuel source by various types of cells. Metabolism of lipids shuttles its energy storage to the core of biochemical reactions and produce energy currency that propels various cellular functions. Conversely, lipids also provide a metabolic flexibility by storing excessive energy in the form of hydrocarbon chain when energy intake exceeds demands and serve as the regulatory factor of cellular energetics (1, 2). The membrane lipids also form the heterogeneous patches of molecular scaffolds that promote the interactions between membrane proteins in signal transduction (1, 3). Lipids and their metabolites are also used as the messengers in various signal transduction cascades (1, 4, 5). The importance of lipids in cancer biology and pathophysiology continues to capture the attention of the biomedical research communities and becomes an actively pursued subject under the “omics”-oriented studies.

Of all the changes in cellular physiology, oxidative glycolysis, among others, stands out as the distinctive phenomenon that hallmarks the metabolic features of the cancer tissues. First reported by Otto Warburg, this phenomenon refers to the predominant shift of cellular glucose metabolism in cancer cells from Krebs cycle-oriented production of energy currency to predominantly glycolysis-based energy currency generation under ample oxygen supply (6, 7, 8, 9). The heightened aerobic glycolysis is intended to meet the elevated metabolic and anabolic demands of the rapidly growing and multiplying cancer cells (8). Conceivably, such a metabolic shift will affect the lipid metabolism of cancer cells and alter the cellular lipid makeup (10, 11, 12). Indeed, cancer-related abnormality in lipid metabolism has been reported since the mid-1950s (13). Nevertheless, detailed lipidomic analysis in cancer cells and tissues has not been realized until the maturation in the analytic platforms and the chemometric tools for systems biology.

Because of its rapid growth and expansion into healthy brain tissue, plus its high probability of recurrence after aggressive treatment approach, the glioma-derived malignant brain tumors almost always inflict significant neurological impairment in the affected subjects in which further treatment is often difficult. The prognosis of such patients varies significantly, spanning from a medium life expectancy of 3 months after conservative treatment, to approximately 15 months following a combined surgical therapy, radiation therapy, and chemotherapy (14). To counteract such an abysmal therapeutic outlook, various intervention approaches, including targeting the metabolic pathways of phospholipids (PLs) (15, 16) and sphingolipids (SLs) (17, 18), have been proposed among other managing strategies.

High frequency of mutation in isocitrate dehydrogenase 1, one of the critical enzymes affecting the cellular lipid metabolism, has been reported in several types of cancers, including glioma that frequently show such a mutation (19). In the revised 2016 World Health Organization classification of tumors of the central nervous system, isocitrate dehydrogenase mutation was included as a critical feature in the classification of astrocytoma, oligodendroglioma, and glioblastoma (20). Deviations of lipidomes in these astrocytic and oligodendroglial tumors from those of the healthy brain tissue are nonetheless expected. Currently, only a few studies explored the changes in lipidome or lipid-associated metabolome in the malignant brain tumors (21, 22, 23, 24, 25, 26). These studies revealed the profile changes in the tumoral diacylglycerols, phosphatidic acids, and FAs (21), reported changes in the tumoral gangliosides, sulfatides, and phosphatidylinositols (PIs) (22), investigated the regulations of phosphatidylglycerols (23), visualized a distance-dependent gradient of lipid-associated metabolome beyond the tumor margin in medulloblastoma (24), associated the metastasis of medulloblastoma with negatively charged PLs (25), and investigated the tissue level of SLs and their metabolites in the glioblastoma (26). Some studies reported the upregulation of lipid-metabolizing enzymes in the tumor microenvironment (27), whereas an earlier study also reported the elevated levels of ether lipids in various types of brain tumors (28). Therefore, the tumoral lipid changes should also be reflected in the common PL classes such as phosphatidylcholines (PCs), phosphatidylethanolamines (PEs), lysophosphatidylcholines (LPCs), lysophosphatidylethanolamines (LPEs), and in the SL classes such as SMs, and ceramides (Cers) that serve as the bulk of the cellular components whose alterations have long been associated with cancer biology (16, 17, 18, 29, 30).

In this study, we investigated the changes of PLs and SLs in the orthotopic mouse glioma to gain insights in their presentation and association with tumoral pathology. A subjective data clustering analysis of MALDI-MSI result permitted the grouping of lipid mass spectrometric features into spatially salient segments in the tumoral brain section and assisted the discovery of several hidden yet significant mass spectrometric features. LC-MS/MS analysis of tumoral lipid information qualitatively and quantitatively corroborated and complemented the MALDI-MSI observation and together provided a more comprehensive snapshot of PLs and SLs in glioma.

## Materials and methods

### Chemicals

MALDI matrix 2,5-dihydroxybenzoic acid was purchased from Alfa Aesar (Lancashire, UK). HPLC grade methanol was purchased from Macron Fine Chemical (Avantor Performance Materials, Central Valley, PA). LC-MS grade acetonitrile and chloroform were purchased from J.T. Baker (Avantor Performance Materials). MS grade ammonium formate was purchased from Sigma-Aldrich Co All the lipid standards were purchased from Avanti Polar Lipids (Alabaster, AL).

### Orthotopic mouse glioma model

The animal care and use were in accordance with the US Public Health Service Policy on Humane Care and Use of Laboratory Animals and approved by the Institutional Animal Care and Use Committees of National Sun Yat-Sen University (no.: 105-05) and Chang Gung Memorial Hospital (no.: 2015032706). Male C57BL6 mice (BioLASCO, Taiwan) and GL261 mouse glioma cell were used to establish the brain tumor model following our previously published protocols (31, 32).

### Collection of the tumoral mouse brains and sample preparations

Mouse brain collection and the preparation of brain sections for MALDI-MSI followed our previously published method (33, 34). In short, the fresh tumoral mouse brains were dissected from the cranium, snap-frozen, cut into 14 μm coronal sections, and immediately vacuum-dried for 20 min. Each tissue section was then drip-washed with 700 μl of 150 mM ammonium acetate solution (34, 35) to ensure a thorough removal of alkali metal cations in tissue. Thereafter, the tissue section was vacuum-dried for another 20 min and then sublimed with the MALDI matrix 2,5-dihydroxybenzoic acid (34, 36) for MALDI-MSI studies.

For LC-MS/MS analyses of brain lipids, the freshly dissected mouse brains were cut into 1 mm coronal brain slices using brain matrices (SA-2175, Alto Stainless Steel Coronal Brain Matrices, Roboz Surgical Instrument Co). The third or fourth slice from the anterior pole of cerebrum usually contained the brain tumor. Tumor brain tissue and its contralateral tumor-free control tissue at the comparable medial-lateral and dorsal-ventral level were individually punctured, weighted, and each placed in a sample vial. A 10-μl aliquot of methanolic internal standard (IS) solution containing 3.25 nmol of 1,2-dimyristoyl-*sn*-glycero-3-phosphoethanolamine (PE 14:0/14:0), 1.88 nmol of 1-myristoyl-2-hydroxy-*sn*-glycero-3-phosphoethanolamine (LPE 14:0), 3.25 nmol of 1,2-dimyristoyl-*sn*-glycero-3-phosphocholine (PC 14:0/14:0), 2.57 nmol of 1-myristoyl-2-hydroxy-*sn*-glycero-3-phosphocholine 14:0 (LPC 14:0), 1.21 nmol of 1,2-dipalmitoyl-*sn*-glycero-3-phospho-(1′-myo-inositol) (PI 16:0/16:0), 1.65 nmol of *N*-(dodecanoyl)-sphing-4-enine-1-phosphocholine (SM d18:1/12:0), and 0.18 nmol of *N*-heptadecanoyl-d-erythro-sphingosine (Cer d18:1/17:0) were added for every 1 mg of brain tissue before homogenization. The amount of the included IS was controlled so to limit the ratio of the peak area from the extracted ion chromatograph of the targeted lipid species to that of its respective IS to between 0.1 and 10 (37).

The weighed brain tissue, approximately 2–3 mg each, was placed in the mixture of 200 μl methanol and 200 μl of 0.1 N HCl in a glass sample vial with a suitable amount of IS solution and 2 μl of antioxidant solution (2,6-di-tert-butyl-4-methylphenol, 10% w/v in methanol) and thoroughly homogenized. After a brief vortex, 800 μl of chloroform was added in the mixture and further vortex-homogenized for 20 min, then centrifuged at 3,000 *g* for 10 min at 10°C (38). The lower organic layer was collected in a new glass sample vial and dried under a gentle stream of nitrogen, sealed with a PTFE insert under a screw cap, and then stored at −80°C until analysis. The dried lipid pellet was dissolved in 800 μl of mobile phase A (see below) immediately before LC-MS/MS analysis.

### Mass spectrometer for MALDI-MSI

The MALDI-MSI studies were carried out on a Bruker Autoflex III MALDI TOF-TOF mass spectrometer equipped with a 355 nm Nd:YAG Smartbeam laser (Bruker Daltonics, Bremen, Germany) operating at 200 Hz, using a laser spot size of “medium.” MALDI-MSI data were collected under positive ion mode with the use of reflectron. The lateral resolution was set to 100 μm for both *x*- and *y*-axes, and ions from 50 consecutive laser shots were summed at each imaging spot (34).

### LC-MS/MS system and relative quantitation of brain PLs and SLs

The LC-MS/MS studies were carried out on a Waters model 2695 separation module coupled with an AmaZon X ion trap mass spectrometer (Bruker Daltonics). Lipid classes were separated by a modified hydrophilic interaction chromatography method (39) on an Ascentis® Express hydrophilic interaction chromatography column (2.1 × 150 mm; particle size 2.7 μm; catalog no.: 53946-U; Supelco) running a gradient elution with the mobile phase delivered at 0.2 ml/min. See “LC-MS/MS mobile phase composition and elution gradient” in Supplemental Data for details.

Eluted PCs, LPCs, and SMs were ionized by the electrospray nozzle under negative ion mode to avoid the interference from the alkali metal cations (40). PIs were also ionized in negative ion mode to enhance their detection. Cers, PEs, and LPEs were ionized in positive ion mode. The precursor ions were isolated with a mass window of ^12^C *m/z* ±0.5 Da for each targeted lipid ions. The mass spectrometer was operated under multiple reaction monitoring mode with the ion charge control set to 100,000 ions and 200 ms. At least 10 data points were obtained along the entire chromatographic peak of each eluted lipid to ensure a proper data representation (41). Three different MS/MS method sets were coupled with the same elution method to examine the lipid species detected by this LC-MS system. In the pilot study, we found that at a proper tissue weight to IS ratio, the variation of relative quantitation would be limited in 10% of the mean value in triplicated measurement. Therefore, each sample was analyzed once with each of the three LC-MS/MS methods with the use of IS depicted above. See supplemental Table S1 for details in LC-MS/MS method sets.

Relative quantitation of PCs, LPCs, and SMs was carried out using the extracted ion chromatographs of the [M + HCOO^−^ - 60]^−^ fragment ions by LC-MS/MS under negative ion mode (42). Quantitation of PIs was performed by acquiring the ion chromatograph of the *m/z* 241.3 fragment ion from the precursor anions (43). The identity of the individual PC, LPC, SM, and PI species was verified by the *m/z* values and the intensities of the FA ions. Quantitation of PEs and LPEs was performed by acquiring the ion chromatographs of the [M + H - 141]^+^ fragment ions from the protonated precursor, whereas the identities of PEs and LPEs were verified by additional LC-MS/MS analyses under negative ion mode. Quantitation of Cers was achieved by acquiring the extracted ion chromatographs of the *m/z* 264.5 fragment ion from the protonated Cer precursors by MS^3^ process (44).

### Data processing and analyses

Unsupervised clustering analyses of MALDI-MSI data were performed using Cardinal MS Imaging Toolbox (Cardinal MSI, version 2.10.0) (45) for R (version 4.1.0) (46) running under Windows 10 operational system with the RStudio (version 1.4.1717, freeware version) as the front end. The MSI data were first converted to Analyze 7.5 file format by FlexImaging software (version 3.0, Bruker Daltonics) for the subsequent processing and analysis in Cardinal MSI. All the data processing, handling, and analyses followed the vignette and instruction accompanied with the software. The segmentation results were exported into TIF image files and rotated and cropped with the GNU Image Manipulation Program (version 2.8.22) when necessary. The spectral features in each clustered segment were exported into text files for further organization.

The MALDI-MS images were extracted by FlexImaging software with a mass selection window of ^12^C ± 0.4 *m/z* of the selected lipid. The extracted ion images were globally normalized to total ion count and exported to TIF image files and then cropped and reoriented using GNU Image Manipulation Program (34).

Relative quantitation of brain lipid species was calculated by the ratio of the chromatographic peak area of the quantitative fragment ion from each lipid to that of the respective IS species. Statistical analyses of LC-MS/MS results were carried out by MetaboAnalyst 5.0 (https://www.metaboanalyst.ca/) (47) using paired comparisons. The sum of each PL and SL class and the sum of all the measured lipids were first analyzed by D'Agostino-Pearson test for distribution normality, followed by the parametric paired *t*-test analysis using GraphPad Prism (version 8.4.3; GraphPad Software, Inc, La Jolla, CA) where *P* < 0.05 was considered statistically significant. The assignment of lipid species follows that of LIPID MAPS (48).

## Results

### Morphology, histology, and the mean MALDI-MSI spectrum of the tumoral mouse brain section

**Fig. 1:**
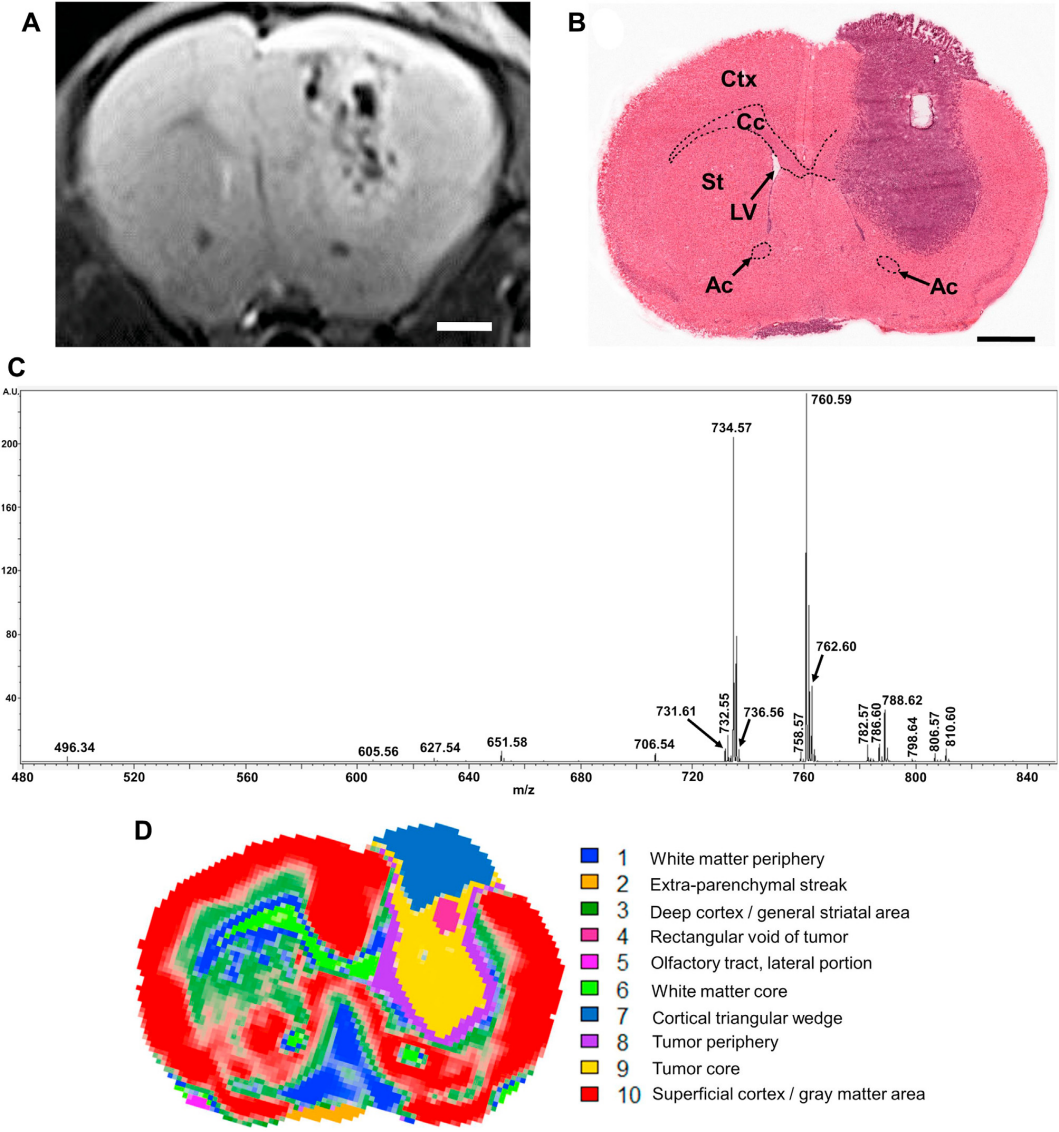
Anatomy, histology, mean MALDI-MSI mass spectrum, and unsupervised clustering of MALDI-MSI data from the coronal mouse brain section containing the orthotopic mouse GL261 glioma. A: T2 enhanced MR image of the in situ mouse glioma 14 days after the implantation of GL261 glioma cells. Bar represents 1 mm. B: H&E-stained image of the coronal mouse brain section after MALDI-MSI data acquisition that yielded the mean MALDI-MSI spectrum in C, and the MALDI images of PC in Figs. 2 and 3, plus the MALDI images of SLs in Fig. 4. Bar represents 1 mm. C: The mean MALDI-MSI mass spectrum of a coronal mouse brain section acquired 15 days after the implantation of GL261 glioma cells. D: Unsupervised clustering and segmentation of the MALDI-MSI data by Spatial Shrunken Centroids clustering method in the Cardinal MS Imaging Toolbox (44). Each of the 10 segments was marked by a unique color and annotated on the right. Ac, anterior commissure; Cc, corpus callosum; Ctx, cortex; LV, lateral ventricle; St, striatum.

The MALDI-MSI study was carried out on three mouse brains containing glioma developed from the GL261 mouse glioma cells implanted into the dorsal striatum. Fig. 1A shows an exemplary T2-enhanced coronal MR image of such a mouse brain tumor containing abundant histological features revealed by MR imaging 14 days after the implantation of mouse glioma cells. A downward tumor growth deep into the ventral striatum and a mushroom cap-like overgrowth above the cortical surface were developed from the implanted tumor cells. This MR image also reveals several heterogeneous patches in the tumor parenchyma, such as the longitudinal area near the medial cortical tumor border, and the quasi-rectangular patch at the cortex-striatum junction slightly lateral to the tumoral midline. A streak-like structure at the bottom of the coronal plane appears to extend leftward from the midline. Fig. 1B shows the image of the H&E-stained brain section after MALDI-MSI data acquisition. The tumor region in the right hemisphere and a small streak-like tissue at the bottom of the brain section left to the midline (the extraparenchymal streak) were stained in blue purple. A compacted hemispheric tumor core appears surrounded by the less compacted tumor periphery bilaterally and ventrally. A longitudinal rectangular void at the cortex-striatum junction slightly lateral to the tumor midline initially presented as a small whitish area that was sloughed off by tissue staining after MALDI-MSI. Since the left hemispheric parenchyma did not appear affected by the tumor in the right hemisphere, it was used as the control for the implanted tumor in this study. Fig. 1C summarizes the mean mass spectrum of MALDI-MSI between *m/z* 480 and 850 of the coronal section in Fig. 1B.

### Unsupervised clustering analysis of MALDI-MSI from the tumoral mouse brain section

The MALDI-MSI dataset was normalized by total ion count, noise filtered, then analyzed with the Spatial Shrunken Centroids algorithm (49) in Cardinal MSI Toolbox (45) running Gaussian weighing. Using 446 distinctive ion peaks, 38 reference ion peaks, a neighborhood smoothing radius (*r*; an arbitrary unit) of 1.5, an initial segment number (*k*) of 15, and a shrinkage parameter (*s*) of 5, the tumoral brain section was clustered into 10 distinctive yet salient segments encompassing the anatomical, pathological, and lipidomic features. Table 1 lists the ^12^C *m/z* value of the ion species and their nonzero *t*-statistic values, which denote their significance in representing each segment when compared with the mass spectrometric features in the MALDI-MSI mean spectrum. The higher the *t*-statistic value, the more significant the lipid species is represented in the segment. The clustering result was crossreferenced with the mouse brain atlas (50, 51) and the previous MS study (34) and concluded into the following segments in Fig. 1D: segment 1 in blue denotes the white matter periphery and the structure in the ventral median brain of the same mass spectrometric features. Segment 6 in lawn green denotes the white matter core in corpus callosum, the anterior commissure, and the bilateral olfactory tracts, whereas the tiny patch of segment 5 in magenta denotes the lateral portion of the olfactory tract. Segment 3 in green marks the deep cortex and the general striatal area. Segment 4 in maroon denotes the tumoral rectangular void deep in the tumor core of segment 9 in yellow. The dorsal portion of the tumor is covered by a cortical triangular wedge of segment 7 in azure. Additional weak segment 7 signal also appears in the tumor core ventromedial to segment 4. Segment 8 in purple denotes the tumor periphery and earmarks the less compacted tumor edges. Segment 2 in orange corresponds to the extraparenchymal streak at the bottom of the section left of midline. Segment 10 in red accounts for the superficial cortex and other gray matter areas not addressed above.

**Table 1:** List of mass spectrometric features (*m/z* values) with nonzero *t*-statistic versus those in the mean MALDI-MSI spectrum in each segment by Spatial Shrunken Centroids method of Cardinal MSI on the MALDI-MSI data of tumoral brain section. See text for details on the interpretation of *t*-statistic value.

Normal brain parenchyma				Tumoral brain parenchyma			
*m/z*	*t*-Statistic	*m/z*	*t*-Statistic	*m/z*	*t*-Statistic	*m/z*	*t*-Statistic
Segment 1				Segment 2			
788.62	31.745	784.60	−0.960	810.60	91.157	731.61	−0.110
760.59	15.208	782.57	−3.761	782.57	75.747	651.58	−1.020
762.60	12.623	736.56	−4.578	808.59	6.496	736.56	−1.610
		706.54	−10.543	784.60	3.986	788.62	−2.398
		758.57	−11.872	627.54	3.027	732.55	−3.855
		732.55	−16.906			760.59	−6.238
		786.60	−17.281			734.57	−8.649
		734.57	−18.160			762.60	−12.453
Segment 3				Segment 4			
762.60	17.405	496.34	−2.339	666.64	95.932	651.58	−0.540
734.57	8.669	784.60	−4.452	638.61	70.556	782.57	−0.650
731.61	4.855	706.54	−19.773	610.58	59.721	810.60	−0.950
651.58	2.887	758.57	−22.169	650.64	51.093	736.56	−2.482
736.56	1.610	732.55	−31.981			788.60	−5.264
		786.60	−39.440			762.60	−16.122
						760.59	−26.966
						734.57	−27.085
Segment 5				Segment 7			
788.62	32.874	732.55	−0.820	786.60	128.083	806.57	−5.314
760.59	2.113	734.57	−13.643	732.55	100.544	731.61	−9.345
				706.54	71.320	651.58	−11.777
				758.57	49.195	810.60	−14.988
				760.59	9.627	782.57	−16.111
				784.60	7.225	736.56	−16.999
				788.62	6.902	762.60	−33.280
				496.34	3.988	734.57	−57.408
				605.56	2.921		
Segment 6				Segment 8			
788.60	62.251	806.57	−0.710	732.55	16.312	651.58	−5.829
605.56	12.262	651.58	−1.850	798.64	15.342	731.61	−5.934
760.59	3.381	706.54	−3.219	786.60	15.323	736.56	−7.968
		758.57	−3.595	496.34	14.221	762.60	−9.963
		731.61	−3.828	782.57	14.114	734.57	−20.532
		732.55	−7.063	760.59	12.058		
		736.56	−8.021	758.57	7.250		
		734.57	−31.684	808.57	3.626		
				772.62	3.243		
				806.57	2.847		
				810.60	2.400		
				788.62	0.450		
				784.60	0.440		
Segment 10				Segment 9			
734.57	88.693	638.61	−0.720	786.60	97.386	806.57	−1.690
736.56	31.095	605.56	−2.111	732.55	93.210	810.57	−7.780
762.60	25.661	666.64	−2.669	758.57	83.100	788.62	−7.914
651.58	18.341	810.60	−2.741	706.59	61.876	731.61	−12.648
731.61	11.231	798.64	−4.436	784.60	28.012	651.58	−15.502
806.57	0.680	496.34	−9.294	496.34	15.878	736.56	−16.418
		784.60	−10.397	798.64	12.049	762.60	−43.317
		760.59	−25.077	760.59	7.426	734.57	−43.529
		706.54	−34.743	772.62	1.170		
		758.57	−39.382				
		788.62	−47.395				
		732.55	−57.759				
		786.60	−71.654				

### MALDI-MSI of PCs in the tumoral mouse brain section

#### Common brain PCs and their analogs

**Fig. 2:**
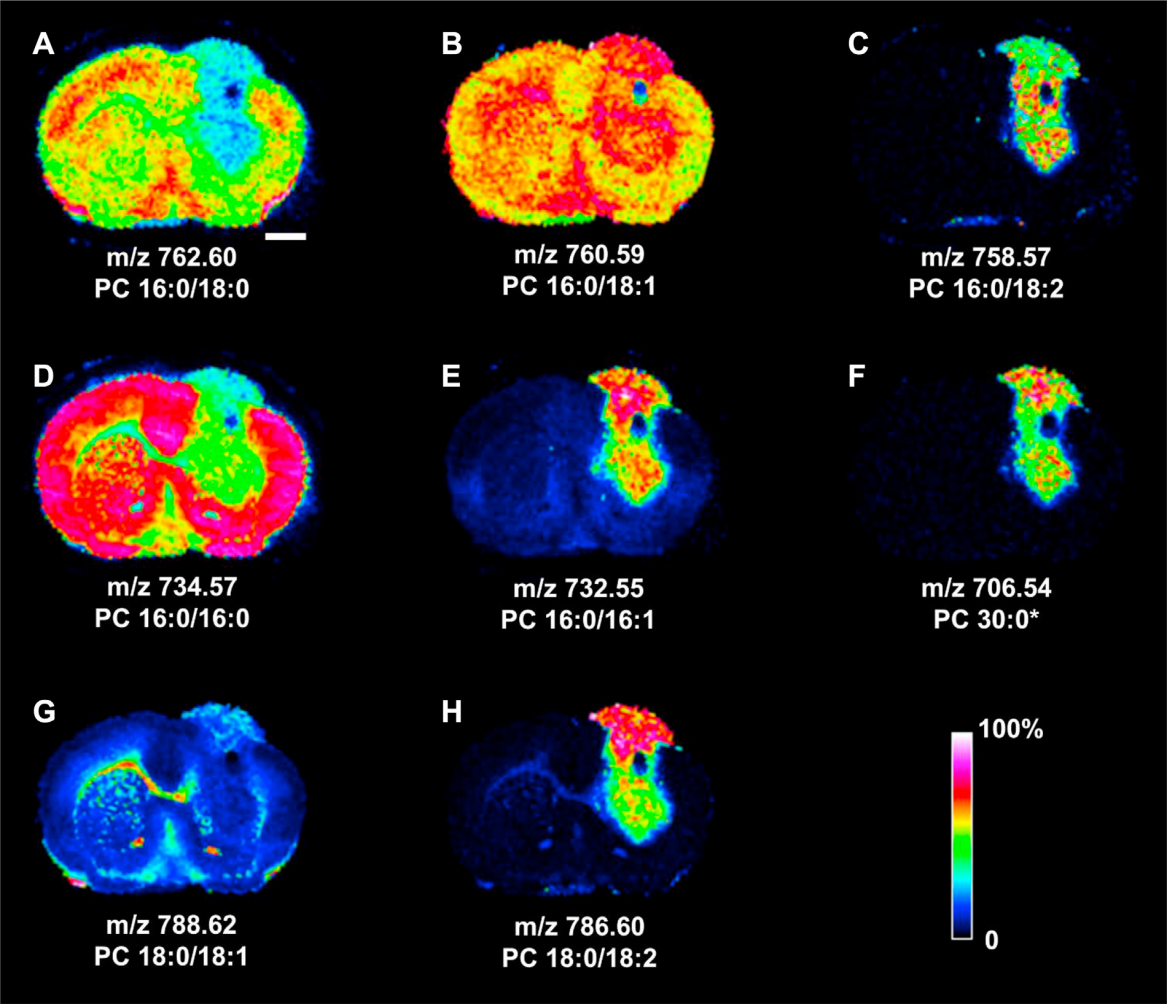
MALDI-MSI of common brain PCs, their analogs, and the diacylglycerol in the tumoral mouse brain section. A: PC 16:0/18:0; B: PC 16:0/18:1; C: PC 16:0/18:2; D: PC 16:0/16:0; E: PC 16:0/16:1; F: PC 30:0; G: PC 18:0/18:1; and H: PC 18:0/18:2. ∗: Identified by *m/z* only. Rainbow bar represents relative abundance scale for the individual lipid image. Bar represents 1 mm. See Fig. 1B for the image of H&E-stained coronal brain section yielding the MALDI images reported in this figure.

Fig. 2 demonstrates the representative MALDI-MSI of common brain PCs in the tumoral brain section. The distributions of PC 16:0/18:0 at *m/z* 762.60 and PC 16:0/16:0 at *m/z* 734.57 were exemplified in Fig. 2A and D, respectively. Both PCs were significantly reduced or even absent in the tumoral regions corresponding to segments 2, 4, 7, 8, and 9. The reduction of PC 16:0/18:0 was more prominent in segments 2, 4, 7, and 9, whereas the reduction of PC 16:0/16:0 appeared homogeneous throughout the tumor and segment 2. PC 16:0/18:1 at *m/z* 760.59, an analog of PC 16:0/18:0 by one additional fatty acyl double bond (FADB), was noticeably increased in segments 7, 8, and 9 (Fig. 2B). PC 16:0/18:2 at *m/z* 758.57, an analog of PC 16:0/18:1 by one additional FADB, accumulated in segments 7 and 9, with emphasis around the tumoral rectangular void, the deep tumoral striatum, and a very mild increase in segment 2 (Fig. 2C). Similarly, PC 16:0/16:1, an analog of PC 16:0/16:0 by one additional FADB at *m/z* 732.55, was seen in segments 7 and 9 (Fig. 2E). This PC was highly abundant yet heterogeneously distributed in segment 7 dorsomedial to the rectangular tumor void.

The tumor growth disrupted the normal callosal distribution of PC 18:0/18:1 at *m/z* 788.62 (Fig. 2G) and distributed in the cortical triangular wedge of segment 7. PC 18:0/18:2 at *m/z* 786.60, an analog of PC 18:0/18:1 by one additional FADB, appeared highly concentrated in segment 7 (Fig. 2H) and moderately upregulated in segment 9 of tumor.

The PC 30:0 at *m/z* 706.54 appeared heterogeneously upregulated in segments 7 and 9 of tumor (Fig. 2F). The tumoral segment 4 that corresponded to the longitudinal rectangular patch in Fig. 1A and the rectangular void in Fig. 1C showed a prominent lack of any PC signals depicted in Fig. 2.

### Polyunsaturated fatty acyl-PCs, LPCs, and ether PCs

**Fig. 3:**
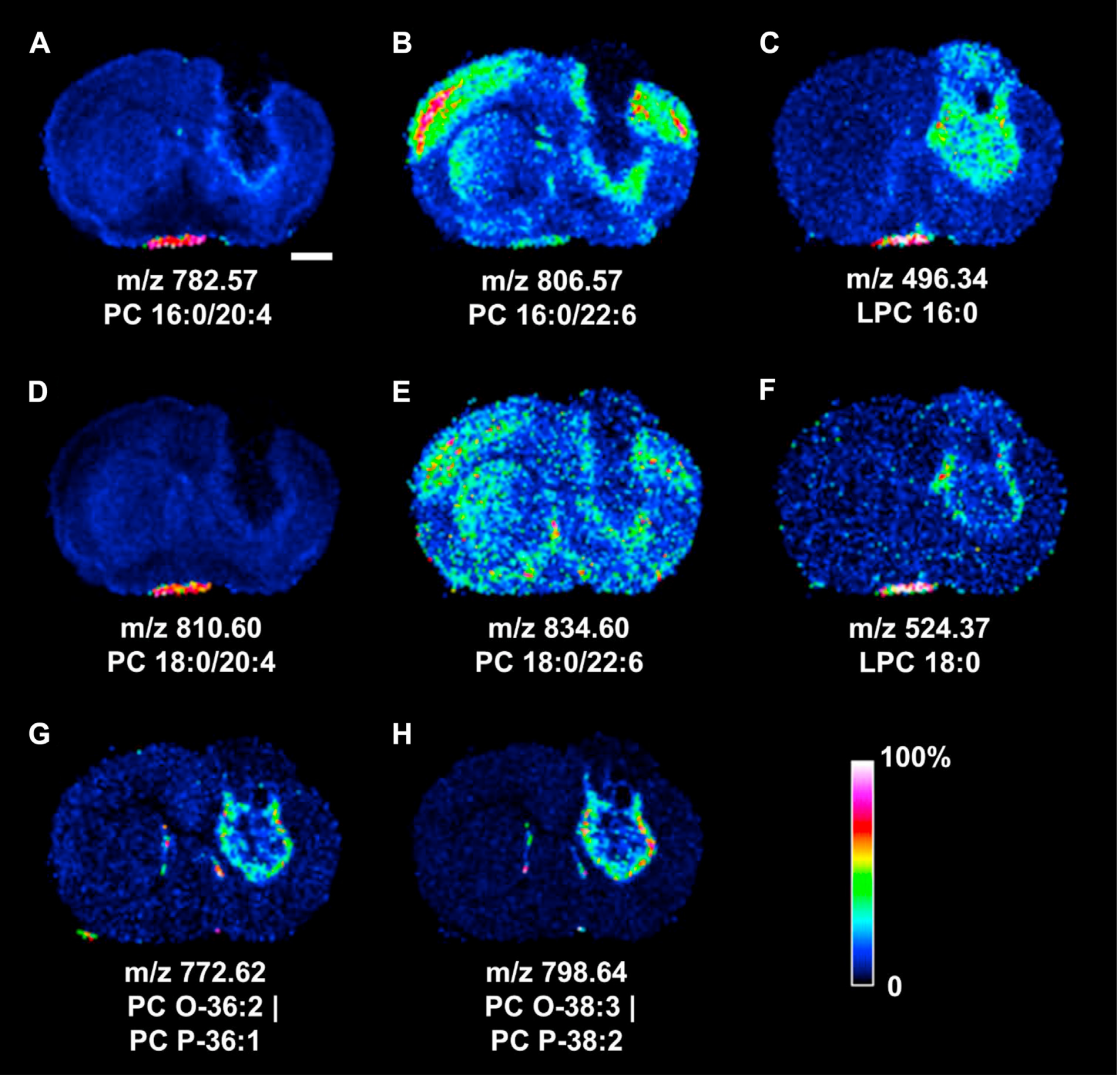
MALDI-MSI of polyunsaturated fatty acyl-PCs, lyso-PCs, and ether PCs in the tumoral mouse brain section. A: PC 16:0/20:4; B: PC 16:0/22:6, C: LPC 16:0; D: PC 18:0/20:4; E: PC 18:0/22:6; F: LPC 18:0; G: PC O (plasmanyl)-36:2|PC P (plasmenyl)-36:1; H: PC O-38:3|PC P-38:2. Rainbow bar represents relative abundance scale for the individual lipid image. Bar represents 1 mm. See Fig. 1B for the image of H&E-stained coronal brain section yielding the MALDI images reported in this figure.

Fig. 3 summarizes the distributions of PUFA PCs, LPCs, and ether PCs in the tumoral mouse brain section. The arachidonyl PC 16:0/20:4 at *m/z* 782.57 (Fig. 3A) was voided in tumoral segments 4, 7, and 9, yet accumulated in tumor periphery at segment 8, and was highly abundant in the extraparenchymal streak in segment 2. This PC also showed a very mild and diffuse increase in lower striatum below the ventral tumor edge. PC 18:0/20:4 at *m/z* 810.60 showed a similar yet less pronounced distribution to that of PC 16:0/20:4 in the tumor periphery of segment 8, yet was prominently present in the extraparenchymal streak in segment 2 (Fig. 3D). The tumor also disrupted the cortical and striatal distribution of DHA-PC 16:0/22:6 at *m/z* 806.57 (Fig. 3B) and PC 18:0/22:6 at *m/z* 834.60 (Fig. 3E). Both DHA-PCs were absent in segments 4, 7, and 9, yet accumulated in the tumoral periphery of segment 8 that was obliquely interrupted at midstriatum level. PC 16:0/22:6 was moderately abundant in segment 2, but PC 18:0/22:6 did not appear to accumulate in this area.

The distributions of LPC 16:0 at *m/z* 496.34 and LPC 18:0 at *m/z* 524.37 were also investigated. LPC 16:0 appeared mostly in segment 8 and 9 at or below the rectangular void. This LPC was haphazardly accumulated in segment 7, yet highly abundant in segment 2 (Fig. 3C). LPC 18:0 was only slightly increased in the medial and lateral tumoral periphery at segment 8 and highly abundant in segment 2 (Fig. 3F).

The unsupervised clustering result surprisingly indicated the *m/z* 772.62 species as one of the representative lipids in the tumor periphery of segment 8 (Table 1) that was absent in the mean MALDI-MSI spectrum (Fig. 1C). This lipid signal, likely originated from the mixture of plasmanyl PC O-36:2 and plasmenyl PC P-36:1 appeared in the lower tumor periphery and alongside the lateral ventricles bilaterally (Fig. 3G). The *m/z* 798.64 signal (Fig. 3H) was likely derived from the mixture of PC O-38:3 and PC P-38:2 and shared a similar distribution pattern to that of *m/z* 772.62. Furthermore, the *m/z* 772.62 signal appeared slightly increased in the olfactory tract in segment 5, whereas both ether lipid signals showed a moderately abundant pinpointed distribution at the medial edge of segment 2.

### MALDI-MSI of SLs in the tumoral mouse brain section

**Fig. 4:**
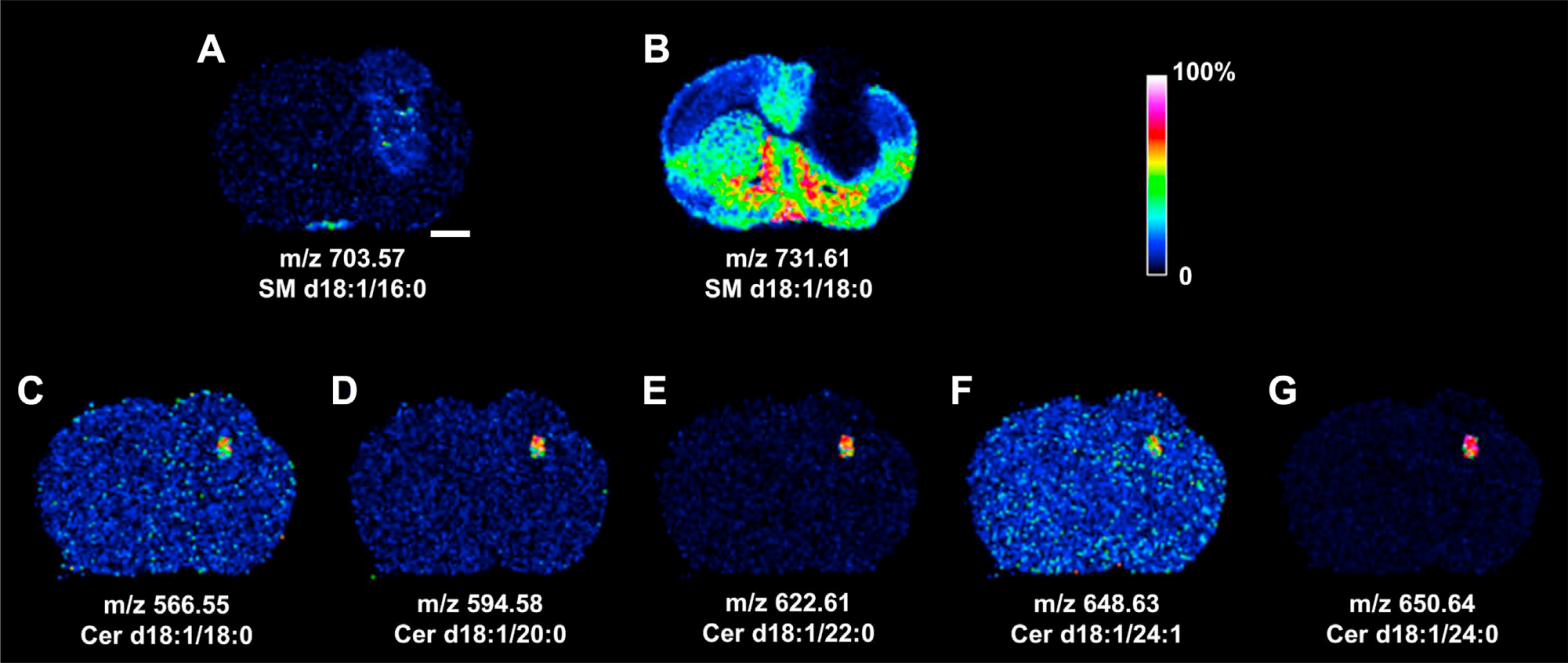
MALDI-MSI of SLs in the tumoral mouse brain section. A: SM d18:1/16:0; B: SM d18:1/18:0; C: Cer d18:1/18:0; D: Cer d18:1/20:0; E: Cer d18:1/22:0; F: Cer d18:1/24:1; G: Cer d18:1/24:0. Rainbow bar represents relative abundance scale for the individual SL image. Bar represents 1 mm. See Fig. 1B for the image of H&E-stained coronal brain section yielding the MALDI images reported in this figure.

Fig. 4 summarizes the MALDI-MSI-detected distributions of SLs in the tumoral brain section. A mild and heterogeneous increase of SM d18:1/16:0 at *m/z* 703.57 in the tumoral segments 2, 7, and 9 is revealed in Fig. 4A. Although not identified as a tumoral feature by the unsupervised clustering analysis, tumoral SM d18:1/16:0 appeared highly upregulated according to the LC-MS/MS measurement (see “LC-MS/MS analyses of PLs and SLs in the tumoral mouse brain” section). The SM d18:1/18:0 at *m/z* 731.61 (Fig. 4B) appeared absent in all tumor-like region and in the white matter areas. Several Cer species were detected in the kernel-like area corresponding to the rectangular void of segment 4 where the distribution of Cer d18:1/18:0 at *m/z* 566.55 (Fig. 4C), Cer d18:1/20:0 at *m/z* 594.58 (Fig. 4D), Cer d18:1/22:0 at *m/z* 622.61 (Fig. 4E), Cer d18:1/24:1 at *m/z* 648.63 (Fig. 4F), and Cer 18:1/24:0 at *m/z* 650.64 (Fig. 4G) was revealed. Table 2 summarizes the MALDI-MSI detected Cer species in segment 4.

**Table 2:** List of Cer species and their *m/z* values detected by MALDI-MSI in segment 4 of Fig. 1D

Cer species detected in segment 4	
[M + H]^+^	Cer species

566.55 ± 0.4	Cer d18:1/18:0
594.58 ± 0.4	Cer d18:1/20:0
622.61 ± 0.4	Cer d18:1/22:0
648.63 ± 0.4	Cer d18:1/24:1
650.64 ± 0.4	Cer d18:1/24:0
554.51 ± 0.4	Cer d18:1/16:0-OH or Cer t18:1(6-OH)/16:0^a^
582.55 ± 0.4	Cer d18:1/18:0-OH or Cer t18:1(6-OH)/18:0^a^
610.58 ± 0.4	Cer d18:1/20:0-OH or Cer t18:1(6-OH)/20:0^a^
638.61 ± 0.4	Cer d18:1/22:0-OH or Cer t18:1(6-OH)/22:0^a^
664.62 ± 0.4	Cer d18:2/24:0-OH^a^
666.64 ± 0.4	Cer d18:1/24:0-OH or Cer t18:1(6-OH)/24:0^a^
584.56 ± 0.4	Cer d18:0/18:0-OH or Cer t18:0/18:0^a^
612.59 ± 0.4	Cer d18:0/20:0-OH or Cer t18:0/20:0^a^
640.62 ± 0.4	Cer d18:0/22:0-OH or Cer t18:0/22:0^a^
668.66 ± 0.4	Cer d18:0/24:0-OH or Cer t18:0/24:0^a^

d18:0 long chain base: dihydrosphingosine; t18:1(6-OH) long chain base; 6-hydroxy sphingosine; t18:0 long chain base: phytosphingosine. Hydroxylation of fatty acyl chain may occur at α, β, or ω position.

a Assignment by *m/z* value only.

Additional MALDI-MSI results of PLs and SLs in the GL261 glioma of mouse brain are provided in Supplemental Fig. S1 for reference.

### LC-MS/MS analyses of PLs and SLs in the tumoral mouse brain

**Fig. 5:**
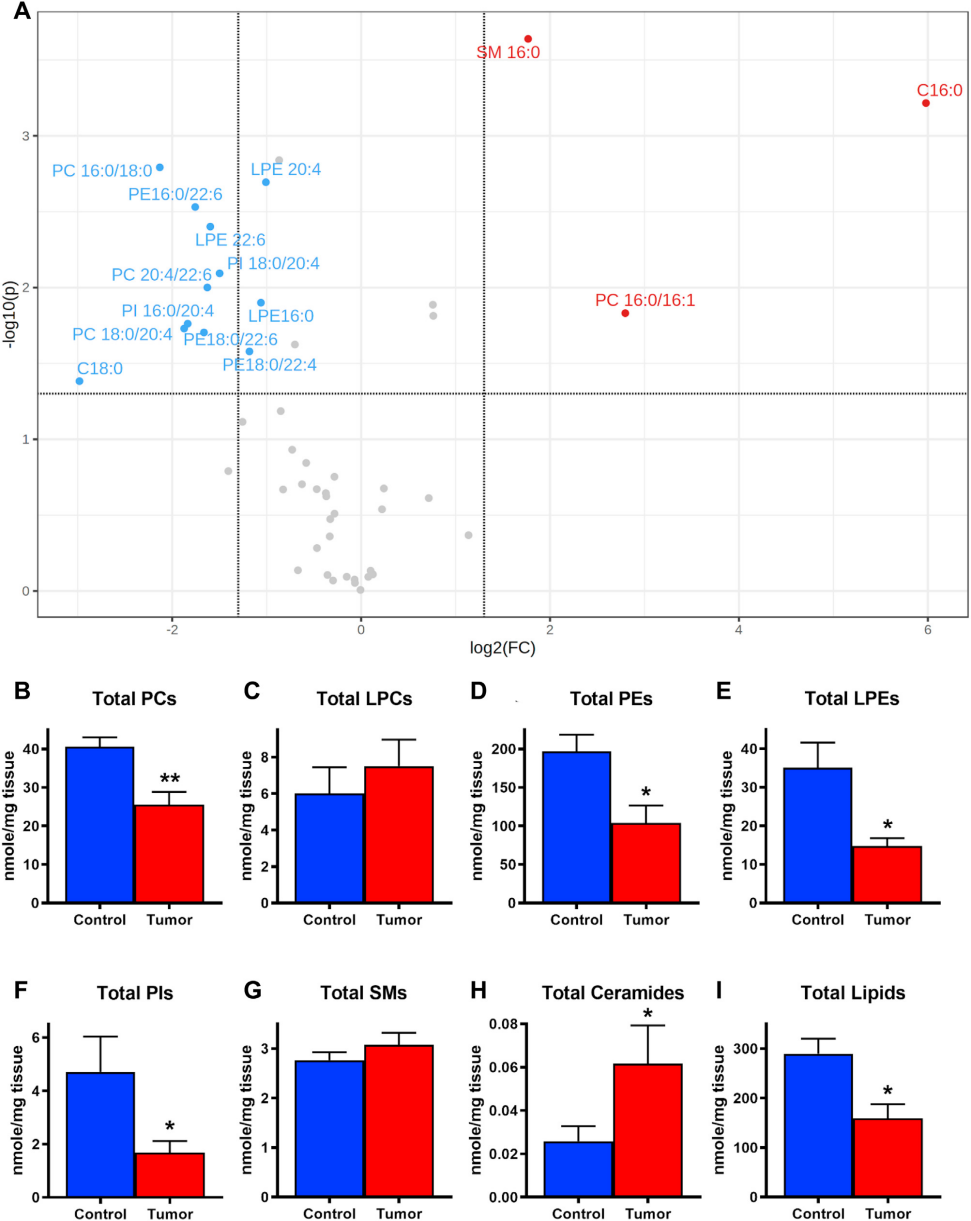
LC-MS/MS analyses of PLs and SLs in the GL261 mouse glioma and the contralateral healthy control brain tissue. A: Volcano plot summarizes the paired *t*-test comparisons of tumoral PLs and SLs to those in the contralateral control brain tissue (N = 8), showing lipid species of statistically significant (*P* < 0.05, −log10(*P*) >1.301; horizontal dash bar) with more than two FCs (i.e., log2(FC) >1 or <−1) in difference. Lipid species marked in red indicates significant increase in tumor, and lipid species marked in blue indicates significant decrease in tumor. Vertical dashed lines denote the log2(FC) cutoff at 1.322 (right, i.e., FC = 2.5) or −1.322 (left; FC = 0.4). Summary of paired *t*-test (mean ± SEM) in B: total PCs; C: total LPCs; D: total PEs; E: total LPEs; F: total PIs; G: total SMs; H: total Cers; and I: total lipids, between the control brain tissue (control; blue bar) and tumor tissue (tumor; red bar). N = 8 each. ∗*P* < 0.05; ∗∗*P* < 0.01. C16:0: Cer d18:1/16:0; C18:0: Cer d18:1/18:0; and SM16:0: SM d18:1/16:0.

The PLs and SLs in the mouse glioma and the contralateral tumor-free control brain tissue were measured by LC-MS/MS. Supplemental Fig. S2 demonstrated the base peak chromatography of PLs and SLs in a control mouse cortex analyzed under negative ion mode LC-MS. The volcano plot in Fig. 5A revealed the tumoral PLs and SLs species showing more than two fold changes (FCs; i.e., log_2_(FC) >1 or <−1) and *P* values of less than 0.05 (i.e., –log10(*P*) >1.301) by paired *t*-test compared with those in the control brain tissue (N = 8). The significant increase of Cer d18:1/16:0 (C 16:0), SM d18:1/16:0 (SM 16:0), and PC 16:0/16:1 (Fig. 5A, marked in red) and the significant decrease of PC 16:0/18:0, PC 18:0/20:4, PC 20:4/22:6, PE 16:0/22:6, PE 18:0/22:6, PE 18:0/22:4, LPE 16:0, LPE 20:4, LPE 22:6, PI 16:0/20:4, PI 18:0/20:4, and Cer d18:1/18:0 (C 18:0) in tumor (Fig. 5A, marked in blue) were revealed. Table 3 lists the tumor lipid species significantly different from that in their control brain tissue by paired *t*-test, along with their *t*-statistics, *P* values, and the −log10(*P*) values. Whether the FC of each listed lipid reached beyond the 2-fold threshold (i.e., log_2_(FC) >1 or <−1) was also denoted in Table 3. The sums of all the measured PCs (total PCs; Fig. 5B), LPCs, (total LPCs; Fig. 5C), PEs (total PEs; Fig. 5D), LPEs (total LPEs; Fig. 5E), PIs (total PIs; Fig. 5F), SMs (total SMs; Fig. 5G), Cers (total Cers; Fig. 5H), and all the measured lipids (total Lipids; Fig. 5I) in tumor were statistically summarized against those in the control brain tissue, respectively. The total tumoral PCs, PEs, LPEs, and PIs appeared significantly less than those in the control brain tissue. The total tumoral LPCs and SMs appeared slightly more than those in the control brain tissue. The significantly more abundant total tumoral Cers was largely contributed by the noticeable increase of Cer d18:1/16:0. Finally, the total lipid content in tumor was significantly less than that in the control brain tissue.

**Table 3:** Summary of the *t*-statistic values, *P* values, and −log10(*P*) values of tumor lipid species statistically different (*P* < 0.05) from those in the control brain tissue (N = 8; paired *t*-test)

Lipid species	*t*-Statistic	*P*	−Log10(*P*)	Log2(FC) > 1 or <−1
SM d18:1/16:0	6.9058	0.0002	3.6380	Yes
Cer d18:1/16:0	5.8853	0.0006	3.2158	Yes
PC 16:0/18:2	3.3079	0.0130	1.8868	No
PC 16:0/16:1	3.2150	0.0148	1.8311	Yes
LPC 16:0	3.1862	0.0154	1.8137	No
Cer d18:1/18:0	−2.4932	0.0414	1.3830	Yes
PE 18:0/22:4	−2.8040	0.0264	1.5788	Yes
SM d18:1/18:0	−2.8772	0.0237	1.6244	No
PE 18:0/22:6	−3.0059	0.0198	1.7039	Yes
PC 18:0/20:4	−3.0481	0.0186	1.7297	Yes
PI 16:0/20:4	−3.1006	0.0173	1.7617	Yes
LPE 16:0	−3.3304	0.0126	1.9002	Yes
PC 20:4/22:6	−3.5001	0.0100	2.0003	Yes
PI 18:0/20:4	−3.6609	0.0081	2.0936	Yes
LPE 22:6	−4.2134	0.0040	2.4013	Yes
PE 16:0/22:6	−4.4572	0.0029	2.5308	Yes
LPE 20:4	−4.7754	0.0020	2.6940	Yes
PC 16:0/18:0	−4.9729	0.0016	2.7921	Yes
PC 16:0/16:0	−5.0698	0.0014	2.8394	No

The FC status of each listed lipids (i.e., log2(FC) >1 or <−1) is also marked.

### Interrogation of gene expression in the human glioma

We interrogated the gene expression of lipid metabolism-related enzymes related to the above observation in the human glioma samples from the database of The Cancer Genome Atlas Research Network (https://www.cancer.gov/tcga). Based on the gene expression analysis result of 20 human glioma samples and 5 normal human brain samples, we found that the FA desaturase 2 (*FADS2*), several phospholipase 2 (*PLA2*) associated with membrane lipid metabolism, two lysophospholipases (*LYPLA*s), PI 4-kinase type 2 beta, two AKT serine/threonine kinases (*AKT*s), *N*-acylsphingosine amidohydrolase 1 (i.e., the acid ceramidase [*ACDase*]), SM phosphodiesterase 2 (i.e., the neutral sphingomyelinase), and sphingosine kinase 1 were upregulated in human glioma (see supplemental Table S2 for details).

## Discussion

In this study, we investigated the changes of common PLs and SLs in the orthotopic mouse glioma using MALDI-MSI and LC-MS/MS techniques. The MR image unveiled several heterogeneous patches in tumor highlighted by different PL and SL species. Unsupervised clustering analysis of MALDI-MSI data stratified the tumoral brain tissue into pathologically salient substructures, revealing region-specific lipid signals, and assisted in the identification of critical lipid species in tumor substructures not seen in the mean MALDI-MSI spectrum. This analysis strategy avoided the bias-prone approach in extracting the ion density maps for tissue lipid distributions. The brain lipid images revealed heterogeneous upregulation and downregulation of tumoral PLs and SLs and substantiated the tumoral substructure stratified by the unsupervised clustering. The lipid information by LC-MS/MS corroborated and supplemented the MALDI-MSI observation. Interrogation of changes in gene expression of enzymes mediating lipid metabolism in human glioma also supported the observed lipidomic results in MALDI-MSI and LC-MS/MS.

The unsupervised clustering analyses of MALDI-MSI data offered an objective approach in clustering the lipid features into spatially salient segments in the tumoral brain section. Several segmentation patterns were obtained from the combination of different weighing methods, neighborhood smooth radius, initial number of segment, and the shrinking parameter. The outcome in Fig. 1D provided a more reasonable depiction of spatial relationship in tumoral lipidomic features associated with the underlying histopathology. Mass features with positive nonzero *t*-statistic values in each segment in Table 1 highlighted the ion species that are systemically enriched to represent the individual segment, whereas ions with negative nonzero *t*-statistic values indicate their under-representation in the individual segment (49).

Both MALDI-MSI and LC-MS/MS results demonstrated the obvious decrease in the common PCs, such as PC 16:0/16:0 and PC 16:0/18:0 in tumor parenchyma. The tissue distributions showed slightly different patterns of decrement for these two PCs in tumor. Parallel to such reduction was the heterogeneous increase of PC 16:0/16:1, PC 16:0/18:1, and PC 16:0/18:2 in tumor. The increase of PC 18:0/18:2 in the cortical triangular wedge was also noted. Such increase of desaturated FA bond(s) in tumor PLs likely reflects the upregulated stearoyl-CoA desaturase activity that introduced the Δ-9 unsaturated bond in palmitoyl and stearoyl FA moieties in the de novo synthesized tumor PCs in bulk and lowered the saturated FA to MUFA ratio (29, 52, 53). Increase of MUFA also modulates the tumorigenic signaling pathways (52, 54), reduces the stress in the endoplasmic reticulum (55), and promotes the survival of tumor cells. Alternatively, the FADS2 may introduce the additional desaturation and increases the number of double bonds at either Δ-9 or Δ-6 position on the FA chain (56). The interrogation of human glioma gene expression showed the significant upregulation of *FADS2*, suggesting the underlying mechanism responsible for this observation. Since the PC 16:0/16:1 and PC 18:0/18:2 shared a largely overlapped distribution in the cortical triangular wedge, a common FA desaturation mechanism coupled with the de novo PL synthesis would account for the upregulation of these two PCs in tumor.

The significant decrease of tumoral PI 16:0/20:4 and PI 18:0/20:4 seen in LC-MS/MS result could be attributed to the upregulation of PI-specific PLC (PI-PLC). Upregulations of PI-PLC and PI 3-kinase, PI 4-kinase, and/or other PI kinases would together activate the downstream Akt/mTOR signaling pathway (57), a commonly altered mechanism highly active in high percentage of glioblastoma (58) that promotes the survival, growth, proliferation, and mobility of tumor cells, resulting in a significant reduction of tumoral PIs. The interrogation of human glioma gene expression revealed the significant upregulation of *AKT*s and PI 4-kinase type 2 beta, suggesting the involvement of upregulated PI-PLC mediating the metabolism of PIs in glioma.

The MALDI-MSI results revealed, in addition to PCs, a significant decrease of tumoral SM d18:1/18:0, indicative of upregulation in tumoral SMase. The SM levels were much lower in several glioma cell lines than those in the normal glial cells (59). However, the immediate downstream product by SMase, such as Cer d18:1/18:0, was only accumulated in the rectangular void deep in the tumor core, marking the area of high apoptotic activity. LC-MS/MS measurement also pointed to an overall reduction in tumoral Cer d18:1/18:0, similar to the previous observation (26). Such precursor-product mismatch suggested the involvement of additional SL metabolic machinery such as ceramidase (CDase) that reduced the tissue Cer and prevented Cer-mediated apoptosis in tumor (18). The downstream product of CDase, i.e., the sphingosine, was likely converted to sphingosine 1-phosphate by the upregulated sphingosine kinase to promote inflammation, survival, growth, oncogenesis, metastasis, stem cell behavior, and microvasculature formation in tumor tissues (26, 60, 61). In one of the studies, it was reported that the sphingosine 1-phosphate tissue level in the glioblastoma was 9-fold higher than that in the control gray matter (26). Indeed, the interrogation of human glioma gene expression indicated upregulation of SM phosphodiesterase 2, N-acylsphingosine amidohydrolase 1, and sphingosine kinase 1, supporting the above protective mechanism to ensure the tumor cell survival. The observed paradoxical increase of tumoral Cer d18:1/16:0 was also similarly reported in the head and neck squamous cell carcinoma (62), breast cancer (63), and glioblastoma (64). The parallel increase of tumoral SM d18:1/16:0 implied the adaptation of yet another SL metabolic mechanism by the tumoral cells to evade the Cer-mediated apoptosis through the formation of the analogous SM species to promote the proliferation, invasion, and immune evasion of the tumor cells (65).

Upregulated tumoral PLA_2_s likely contributed to the increased LPCs, which in turn may be subjected to two likely downstream metabolic fates. Upregulation of autotaxin might convert LPCs to lysophosphatidic acids and promote the survival and mobility of the malignant brain tumor cells (30). Activation of LYPLAs that process and release the FA chains of the LPCs and shuttled them for the de novo PL synthesis (66) might be the alternative metabolic fate of the increased LPCs. Interrogation into human glioma gene expression revealed the upregulation of several *PLA2* subtypes and two *LYPLA*s, supporting the likely fates of the increased tumoral LPCs, and pointed to another likely pathway of lipid metabolism involved with the progression of glioma.

Elevated tissue level of choline ether PLs in glioblastomas, astrocytomas, acoustic neurinoma, oligodendroglioma, and meningioma was reported in an early study of lipid composition in brain tumors (28). Later investigation further indicated the upregulation of alkyl glycerone phosphate synthase, the critical ether lipid synthesis enzyme, in various types of cancer cells. Knockdown of alkyl glycerone phosphate synthase significantly reduced the content of ether lipids, FAs, eicosanoids, acyl PLs, and impaired the cellular migration and invasion of cancer cells (67). Lipid imaging in this study revealed a trace amount of ether PCs in the periphery of deep striatal glioma and along the right and left lateral ventricles. The quasi-spherical wrapping of the striatal tumor by ether PCs marked the likely frontiers of tumor expansion that, by reaching the right lateral ventricle, disseminated to the left lateral ventricle and other remote locations.

The PL and SL profiles of the extraparenchymal streak appeared similar but not identical to those in the hemispheric tumor. The upregulated PUFA-PCs, LPCs, and SM in this area were biochemically analogous to the translocated tumor tissue containing higher levels of PCs and other lipids (68). Careful consultation with the mouse brain atlas (51) concluded that this structure anomaly was likely derived from the translocated tumor cells in the primary tumor site through the cerebral ventricle system to this diencephalic location juxtapose to the third ventricle.

Together, the common PL and SL profiles in the orthotopic mouse glioma revealed several altered features in lipid metabolism associated with the underlying signaling pathways that promote the survival, maintenance, proliferation, migration, and invasion of different tumor cell populations and ensure their progression and expansion. These parallel cellular mechanisms of tumoral lipid metabolism underscore the need in developing therapeutic strategies concurrently targeting multiple mechanisms of cell survival, proliferation, and invasion of glioma for an effective management of such a deleterious CNS malignancy.

## Data Availability

The data that support the findings of this study are available from the corresponding author upon reasonable request.

## Supplemental Data

This article contains supplemental data.

## Conflict of Interest

The authors declare that they have no conflicts of interest with the contents of this article.
