# Supplementary material for: A mass spectrometry imaging and lipidomic investigation reveals aberrant lipid metabolism in the orthotopic mouse glioma
Source: J Lipid Res. 2022 Oct 20;63(12):100304. doi: 10.1016/j.jlr.2022.100304 (PMC9761856; doi:10.1016/j.jlr.2022.100304)
Supplement: Supplementary Tables and Figure [file mmc1.docx]

**Supplementary Information**

**A Mass Spectrometry Imaging and Lipidomic Investigation Reveals Aberrant Lipid Metabolism in the Orthotopic Mouse Glioma**

Hay-Yan J. Wang^1^*^#^, Chiung-Yin Huang^2,3#^, Kuo-Chen Wei^2,3,4,5^, Kuo-Chen Hung^6^

^1^Department of Biological Sciences, National Sun Yat-Sen University, Kaohsiung, Taiwan

^2^Neuroscience Research Center, Chang Gung Memorial Hospital, Taoyuan, Taiwan

^3^Department of Neurosurgery, New Taipei Municipal TuCheng Hospital, New Taipei City, Taiwan

^4^Department of Neurosurgery, Chang Gung Memorial Hospital, Taoyuan, Taiwan

^5^School of Medicine, Chang Gung University, Taoyuan, Taiwan

^6^Department of Surgery, Kaohsiung Chang Gung Memorial Hospital, Kaohsiung, Chang Gung University College of Medicine, Taiwan

*: Correspondence:

Hay-Yan J. Wang, Ph.D.

Associate Professor, Department of Biological Sciences

National Sun Yat-Sen University

70 Lien-Hai Rd. Kaohsiung 80424, Taiwan

Email: [hyjwang@mail.nsysu.edu.tw](mailto:hyjwang@faculty.nsysu.edu.tw)

Tel: +886-7-525-3601

ORCID iD: <https://orcid.org/0000-0002-8373-5138>

#: These authors contributed equally to this work.

**LC-MS/MS mobile phase composition and elution gradient**

Lipid classes were separated by a modified hydrophilic interaction chromatography (HILIC) method on an Ascentis^®^ Express HILIC column (2.1 × 150 mm; particle size 2.7 µm, Cat. No. 53946-U, Supelco). Mobile phase A was composed of 85% (v/v) ACN, 10% MeOH, and 5% H_2_O, and the mobile phase B was composed of 65% ACN, 10% MeOH, and 25% H_2_O. Both mobile phases contained 0.04% (v/v) formic acid and 1mM of ammonium formate. The mobile phase was delivered at 0.2 mL/min, using the following linear gradient: 90% A from 0-6 min, then linearly decreased to 70% A from 6-10 min, followed by further linear decrease to 50% A from 10-16 min, then held at 50% A from 16-20 min, and returned to 90% A from 20-20.1 min, and held at 90% till the end of the run at 35 min. The chromatograph of the 7 monitored lipid classes was exemplified by the base peak chromatography under negative ion mode in Supplementary Figure s1 below.


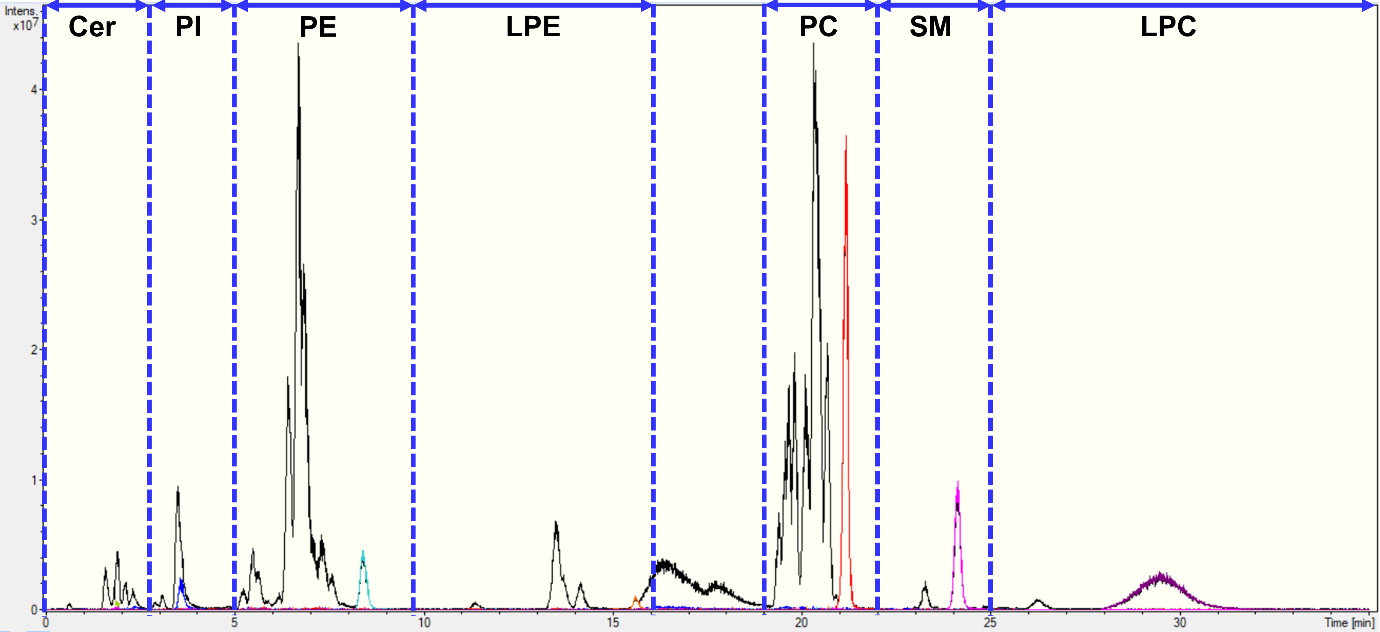


**Supplementary Figure S1**: LC-MS base peak chromatography of PLs and SLs from the normal control mouse cortex. Vertical dash lines delineate the chromatography segments of eluted lipid classes. The colored chromatographic tracing overlapping the base peak chromatograph in each elution segment denotes the extracted ion chromatograph of the internal standard of each lipid class. In the early elution segments co-elution of more abundant lipids other than the targeted lipid class may occur, potentially leading to the misinterpretation of the overall signal-to-noise ratio.


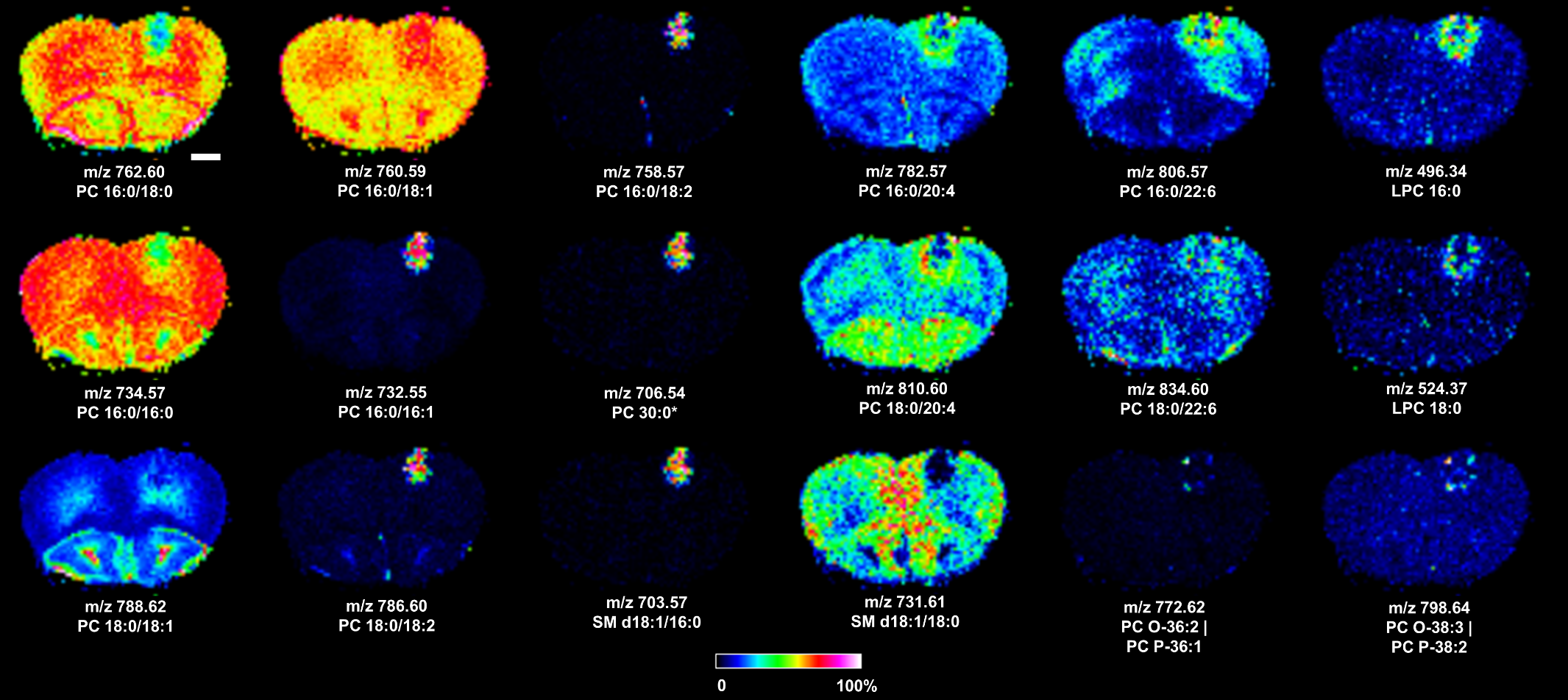


**Supplementary Figure S2:** Additional MALDI-MSI of GL261 glioma in mouse brain derived from surgical injection of the GL261 mouse glioma cells 15 days after the implantation procedure. See text for the method details. All the panel legends were identical to their respective panels in Figure s2, 3, and 4. *: Lipid identified by m/z value only. The nature of lipid expression in the tumor parenchyma appeared highly similar, if not identical, to their respective species in Figures 2, 3, and 4. Bar = 1mm. Rainbow bar: Relative abundance scale for the individual lipid image.

| **LC-MS/MS Method 1** | | | | | |  | |  | |  | |
| --- | --- | --- | --- | --- | --- | --- | --- | --- | --- | --- | --- |
| Lipid Class and Species | | | | Precursor ion (m/z) | | Fragment ion (m/z) | | Peak retention time (min) | | Collision voltage (V) | |
| Ceramide (Cer) | | Cer d18:1/17:0 (IS) | | 552.5 | | 264.5 | | 1.9 | | 0.95 | |
|  |  | Cer d18:1/18:0 | | 566.5 | | 264.5 | | 1.9 | |  |  |
|  |  | Cer d18:1/20:0 | | 594.5 | | 264.5 | | 1.9 | |  |  |
|  |  | Cer d18:1/22:0 | | 622.5 | | 264.5 | | 2.7 | |  |  |
| PI | | PI 16:0/16:0 (IS) | | 809.6 | | 241.3 | | 3.8 | | 0.7 | |
|  |  | PI 16:0/20:4 | | 857.6 | | 241.3 | | 3.7 | |  |  |
|  |  | PI 18:0/20:4 | | 885.6 | | 241.3 | | 3.6 | |  |  |
| PE | | PE 14:0/14:0 (IS) | | 636.5 | | 495.5 | | 8.5 | | 0.5 | |
|  |  | PE 16:0/18:1 | | 718.6 | | 577.6 | | 7.7 | |  |  |
|  |  | PE 16:0/20:4 | | 740.6 | | 599.6 | | 7.2 | |  |  |
|  |  | PE 18:0/18:2 | | 744.6 | | 603.6 | | 7.4 | |  |  |
|  |  | PE 18:0/20:5 | | 766.6 | | 625.6 | | 7.0 | |  |  |
| LPE | | LPE 14:0 (IS) | | 426.5 | | 408.5 | | 15.7 | | 0.8 | |
|  |  | LPE 16:0 | | 454.3 | | 313.3 | | 14.9 | |  |  |
|  |  | LPE 18:1 | | 480.3 | | 339.3 | | 14.4 | |  |  |
|  |  | LPE 18:0 | | 482.3 | | 341.3 | | 14.5 | |  |  |
|  |  | LPE 20:4 | | 502.3 | | 361.3 | | 14.1 | |  |  |
| PC | | PC 14:0/14:0 (IS) | | 722.6 | | 662.6 | | 21.3 | | 1.0 | |
|  |  | PC 16:0/18:1 | | 804.6 | | 744.6 | | 20.3 | |  |  |
|  |  | PC 16:0/20:4 | | 826.6 | | 766.6 | | 19.8 | |  |  |
|  |  | PC 18:0/18:1 | | 832.6 | | 772.6 | | 20.3 | |  |  |
|  |  | PC 16:0/22:6 | | 850.6 | | 790.6 | | 19.9 | |  |  |
|  |  | PC 20:4/22:6 | | 898.6 | | 838.6 | | 19.1 | |  |  |
| SM | | SM d18:1/12:0 (IS) | | 691.5 | | 631.5 | | 24.2 | | 1.0 | |
|  |  | SM d18:1/16:0 | | 747.6 | | 687.6 | | 24.0 | |  |  |
|  |  | SM d18:1/18:1 | | 773.6 | | 713.6 | | 23.9 | |  |  |
|  |  | SM d18:1/18:0 | | 775.6 | | 715.6 | | 23.8 | |  |  |
|  |  | SM d18:1/20:0 | | 803.6 | | 743.6 | | 23.5 | |  |  |
|  |  | SM d18:1/24:1 | | 857.6 | | 797.6 | | 23.0 | |  |  |
| **LC-MS/MS Method 2** | | | | |  | |  | |  | |  |
| Lipid Class and Species | | | Precursor ion (m/z) | | Fragment ion (m/z) | | Peak retention time (min) | | Collision voltage (V) | |  |
| PE | PE 14:0/14:0 (IS) | | 636.5 | | 495.5 | | 8.5 | | 0.5 | |  |
|  | PE 18:0/22:4 | | 796.6 | | 655.6 | | 6.9 | |  |  |  |
| PC | PC 14:0/14:0 (IS) | | 722.6 | | 662.6 | | 21.3 | | 1.0 | |  |
|  | PC 14:0/16:0 | | 750.6 | | 690.6 | | 21.0 | |  |  |  |
|  | PC 16:0/16:1 | | 776.6 | | 716.6 | | 21.2 | |  |  |  |
|  | PC 16:0/16:0 | | 778.6 | | 718.6 | | 21.0 | |  |  |  |

**(Continued)**

| **LC-MS/MS Method 3** | | |  |  |  |
| --- | --- | --- | --- | --- | --- |
| Lipid Class and Species | | Precursor ion (m/z) | Fragment ion (m/z) | Peak retention time (min) | Collision voltage (V) |
| Ceramide (Cer) | Cer d18:1/17:0 (IS) | 552.5 | 264.5 | 1.9 | 0.95 |
|  | Cer d18:1/16:0 | 538.5 | 264.5 | 1.9 |  |
|  | Cer d18:1/18:1 | 564.5 | 264.5 | 2.2 |  |
|  | Cer d18:1/24:0 | 650.5 | 264.5 | 2.2 |  |
| PI | PI 16:0/16:0 (IS) | 809.6 | 241.3 | 3.8 | 0.7 |
|  | PI 18:1/20:4 | 883.6 | 241.3 | 3.7 |  |
| PE | PE 14:0/14:0 (IS) | 636.5 | 495.5 | 8.5 | 0.5 |
|  | PE 18:0/18:1 | 746.6 | 605.6 | 7.4 |  |
|  | PE 16:0/22:6 | 764.6 | 623.6 | 7.0 |  |
|  | PE 18:0/20:4 | 768.6 | 627.6 | 7.0 |  |
|  | PE 18:1/22:6 | 790.6 | 649.6 | 6.8 |  |
|  | PE 18:0/22:6 | 792.6 | 651.6 | 6.8 |  |
| LPE | LPE 14:0 (IS) | 426.5 | 408.5 | 15.7 | 0.8 |
|  | LPE 20:1 | 508.3 | 367.3 | 14.1 |  |
|  | LPE 22:6 | 526.3 | 385.3 | 13.8 |  |
|  | LPE 22:4 | 530.3 | 389.3 | 13.7 |  |
| PC | PC 14:0/14:0 (IS) | 722.6 | 662.6 | 21.3 | 1.0 |
|  | PC 16:0/18:2 | 802.6 | 742.6 | 21.0 |  |
|  | PC 16:0/18:0 | 806.6 | 746.6 | 20.8 |  |
|  | PC 18:0/18:2 | 830.6 | 770.6 | 20.0 |  |
|  | PC 18:0/20:4 | 854.6 | 794.6 | 19.7 |  |
|  | PC 18:0/22:6 | 878.6 | 818.6 | 19.7 |  |
| LPC | LPC 14:0 (IS) | 512.3 | 452.3 | 29.5 | 1.0 |
|  | LPC 16:0 | 540.3 | 480.3 | 26.0 |  |
|  | LPC 18:1 | 566.3 | 506.3 | 25.4 |  |
|  | LPC 18:0 | 568.3 | 508.3 | 26.4 |  |
|  | LPC 20:4 | 588.3 | 528.3 | 25.1 |  |
|  | LPC 22:6 | 612.3 | 552.3 | 24.8 |  |

**Supplementary Table S1:** Three LC-MS/MS method set and the lipid species, the m/z values of the precursor and the fragment ions, the peak retention times, and the collisional voltages used for the quantitation of brain lipid species eluted by the same chromatographic method. IS: internal standard.

| **Gene abbreviation** | **NCBI gene ID** | **Gene name** | **Expression Fold Change vs. control** |
| --- | --- | --- | --- |
| *FADS2* | 9415 | fatty acid desaturase 2 | 2.434 |
| *SCD5* | 79966 | stearoyl-CoA desaturase 5 | 0.516 |
| *SCD* | 6319 | stearoyl-CoA desaturase | 0.497 |
| *PLA2G2A* | 5320 | phospholipase A2 group IIA | 79.096 |
| *PLA2G5* | 5322 | phospholipase A2 group V | 11.485 |
| *PLA2G2D* | 26279 | phospholipase A2 group IID | 6.103 |
| *PLA2G1B* | 5319 | phospholipase A2 group IB | 2.031 |
| *LYPLA1* | 10434 | lysophospholipase 1 | 5.414 |
| *LYPLA2* | 11313 | lysophospholipase 2 | 1.734 |
| *PI4K2B* | 55300 | phosphatidylinositol 4-kinase type 2 beta | 3.441 |
| *AKT1* | 207 | AKT serine/threonine kinase 1 | 1.809 |
| *AKT2* | 208 | AKT serine/threonine kinase 2 | 1.569 |
| *ASAH1* | 427 | N-acylsphingosine amidohydrolase 1 | 1.906 |
| *SMPD2* | 6610 | sphingomyelin phosphodiesterase 2 | 1.691 |
| *SPHK1* | 8877 | sphingosine kinase 1 | 1.685 |
| *SPHK2* | 56848 | sphingosine kinase 2 | 0.716 |

**Supplementary Table S2:** Gene expression interrogation of lipid metabolism-related enzymes in human glioma sample published by the TCGA Research Network (<https://www.cancer.gov/tcga>). The expression fold change was calculated from the results of 20 human glioma samples and 5 normal human brain samples.
